# Supplementary material for: MetaRibo-Seq measures translation in microbiomes
Source: Nat Commun. 2020 Jun 29;11:3268. doi: 10.1038/s41467-020-17081-z (PMC7324362; doi:10.1038/s41467-020-17081-z)
Supplement: Supplementary file 10 — Supplementary Data 7 [file 41467_2020_17081_MOESM10_ESM.zip › File2/Confidence_High_Taxonomy/35980_out.krona.html]

Javascript must be enabled to view this page.

members
magnitude
magnitudeUnassigned
count
unassigned
taxon
rank

35980\_out

1

2
superkingdom
1

1
phylum
1224

68525
subphylum
1

1
29547
class

213849
order
1

72294
family
1

194
genus
1


SRS016474\_contig\_number\_contig-100\_3327.37227
1
199
species
